# Rutin inhibited the advanced glycation end products-stimulated inflammatory response and extra-cellular matrix degeneration via targeting TRAF-6 and BCL-2 proteins in mouse model of osteoarthritis

**DOI:** 10.18632/aging.203470

**Published:** 2021-09-22

**Authors:** Xiang Chen, Mingchuan Yu, Wei Xu, Linfeng Zou, Jing Ye, Yu Liu, Yuhong Xiao, Jun Luo

**Affiliations:** 1 Department of Rehabilitation Medicine, The Second Affiliated Hospital of Nanchang University, Nanchang 330000, Jiangxi Province, China

**Keywords:** osteoarthritis, TRAF-6, BCL-2, NF-κB/MAPK

## Abstract

Background: Osteoarthritis (OA) is degenerative joint disorder mainly characterized by long-term pain with limited activity of joints, the disease has no effective preventative therapy. Rutin (RUT) is a flavonoid compound, present naturally. The flavonoid shows range of biological activities such as anti-inflammatory and anti-cancer effect. We screened RUT for its activity against osteoarthritis with *in vivo* and *in vitro* models of osteoarthritis.

Methods: Animal model of OA was developed using C57BL/6 mice by surgical destabilization of medial meniscus. For *in vitro* studies the human articular cartilage tissues were used which were collected from osteoarthritis patients and were processed to isolate chondrocytes. The chondrocytes were submitted to advanced glycation end products (AGEs) for inducing osteoarthritis *in vitro*. Cell viability was done by CCK-8 assay, ELISA analysis for MMP13, collage II, PGE2, IL-6, TNF-α, ADAMTS-5 and MMP-13. Western blot analysis was done for expression of proteins and *in silico* analysis was done by docking studies.

Results: Pretreatment of RT showed no cytotoxic effect and also ameliorated the AGE mediated inflammatory reaction on human chondrocytes *in vitro*. Treatment of RT inhibited the levels of COX-2 and iNOS in AGE exposed chondrocytes. RT decreased the AGE mediated up-regulation of IL-6, NO, TNF-α and PGE-2 in a dose dependent manner. Pretreatment of RT decreased the extracellular matrix degradation, inhibited expression of TRAF-6 and BCL-2 the NF-κB/MAPK pathway proteins. The treatment of RT in mice prevented the calcification of cartilage tissues, loss of proteoglycans and also halted the narrowing of joint space is mice subjected to osteoarthritis. The *in-silico* analysis suggested potential binding affinity of RT with TRAF-6 and BCL-2.

Conclusion: In brief RT inhibited AGE-induced inflammatory reaction and also degradation of ECM via targeting the NF-κB/MAPK pathway proteins BCL-2 and TRAF-6. RT can be a potential molecule in treating OA.

## INTRODUCTION

Osteoarthritis (OA) is reported to be one of the major factors responsible for disability of old age worldwide [1, 2]. However, the treatment approaches in countering OA are very limited with some therapeutic molecules or surgeries, however these approaches provide only a limited control over development and progression of OA [3, 4]. In addition to this, studies have emerged which make use of alternate approaches such as physical activity (Physiotherapy), transplantation of stem cells which promote restoration of cartilages in animal models of OA [5–8]. Presently, the pathogenesis and etiology responsible for OA are still not clearly understood, however aging is one of the factors responsible for development and progression of OA [9, 10]. Millard reaction which is the non-enzymatic glycation of macromolecules leads to production of advanced glycation end products (AGEs) [11–13]. This leads to over accretion of AGEs in the cartilages of joints which affects the mechanical activity of cartilages and leads to imbalance between the anabolism, catabolism and extracellular matrix molecule (ECM) [14, 15]. Additionally, the receptor of AGE (RAGE) has been reported to be present in the synoviocytes and articular chondrocytes [16]. The AGEs activate RAGE in the chondrocytes and results in generation of catabolic mediators and pro-inflammatory factors such as matrix metalloproteases (MMPs), interleukin, TNF-α and A Disintegrin and Metalloproteinase with Thrombospondin motifs (ADAMTS), which leads to dysfunction of chondrocytes and degeneration of ECM [17].

It has been reported that TRAF-6 and BCL-2 proteins are closely associated with inflammatory disorder, such as osteoarthritis [18, 19]. Levels of both BCL-2 and TRAF-6 proteins are elevated in inflammatory disorder conditions such as osteoarthritis and rheumatoid arthritis [18, 19]. Also, BCL-2 and TRAF-6 proteins are capable of activating the NF-κB/MAPK pathway [20, 21]. In addition to this AGEs have been reported to increase the levels of TRAF-6 [22] and BCL-2 [23] both of which are the important members of mitogen-activated protein kinase (MAPK) and Nuclear factor kappa-B (NF-κB) family proteins, hence MAPK and NF-κB cascades are regarded as potential targets for treating OA.

Rutin (RT) is a natural flavonoid compound; it is widely distributed in fruits, vegetables and wheat [24]. RT has been found to show a range of biological activities such as cardiovascular diseases, cerebrovascular disorders, virus infection, inflammatory conditions and also as antioxidant [25]. Rutin has been reported to prevent LPS induced inflammation by targeting the TRAF-6 associated signaling pathway [26]. In a study RT ameliorated inflammatory condition in arthritis condition by targeting the various inflammatory proteins including BCL-2 [27]. RT attenuated LPS mediated inflammation in muscle cells by inhibiting the levels of IL-6 both at protein and mRNA levels [28]. However, the role of this potential bioactive compound RT in osteoarthritis remains unexplored, looking into the bioactive potential of RT we postulated that it may exert protective effect on chondrocytes against development of osteoarthritis. In the present work, *in vitro* we exposed the human chondrocytes to AGEs. For *in vivo* studies, we developed an animal model of OA by surgical method for assessing the role and the potential mechanism of RT in OA. We also performed *in silico* molecular docking analysis for confirming the affinity of RT with target protein for supporting the *in vitro* and *in vivo* findings.

## MATERIALS AND METHODS

### Reagents and antibodies

Rutin (purity > 98.5%) was procured from Sigma Aldrich USA. The primary antibodies for JNK, p-JNK, p65, p38, p-p38, COX2 and IκBα were procured from cell signaling tech. USA. Antibodies for collagen II, iNOS, GAPDH, Lamin B and ADAMTS5 were obtained from Abcam USA. The anti-mouse and anti-rabbit antibodies were procured from Abcam USA. Safranin-O, type II collagenase, Fast green, Bovine serum albumin and AGE-Bovine serum albumin were procured from Thermo Fisher USA. All the cell culture reagents were bought from Sigma Aldrich USA.

### Animal model of osteoarthritis

For establishing the *in vivo* model of OA we selected C57BL/6 male wild type mice, the animals were obtained from the preclinical department of The Second Affiliated Hospital of Nanchang University, China. The animal protocols were approved by the animal ethical review board of The Second Affiliated Hospital of Nanchang University, China (approval number NU002AC). The animal experiments were in agreement to the guidelines of ethical review of laboratory animal welfare People’s Republic of China National Standard GB/T 35892-2018. For inducing OA in mice, the mice were submitted to surgical destabilization of the medial meniscus (DMM) as discussed earlier with minor modifications wherever needed [25]. Briefly, the mice were anesthetized by pentobarbital anesthesia (40 mg/kg), the joint capsule of knee (right) was operated, a small incision was made with the help of microsurgical scissor on the medial region of patellar tendon and the meniscotibial ligament. The sham operated mice were operated for arthrotomy devoid of incision on the ligament. The animals were divided into 3 groups, the sham operated mice, the DMM operated mice and DMM operated + RT treated mice.

### Culture of primary human chondrocytes

The human articular cartilage tissues were collected from 10 patients diagnosed for osteoarthritis at ‘The Second Affiliated Hospital of Nanchang University, China’ aging between 60-70 years (5 men and 5 women), the patients were undergoing operation for total knee replacement. All the experiments were done in accordance guidelines from Declaration of Helsinki and were approved by human ethical review board of The Second Affiliated Hospital of Nanchang University, China, the approval number was BC102A. Prior to collection of articular cartilage tissues, informed consents were signed by the subjects involved in the study. The obtained tissues were divided into small pieces and rinsed thrice with phosphate buffered saline, the small pieces were treated with collagenase II (0.2%) for digesting the cartilage tissues at 25° C for 6 hours. The chondrocytes obtained were then cultured in the tissue culture flasks having DMEM media added with fetal bovine serum (10%) and Streptomycin (1%) at 25° C with 5% CO2. When the chondrocytes reached confluence of 80%, they were submitted to be starved in media free of serum for 12 hours. The cells (5 × 10^5^) were incubated in culture plates. The chondrocytes of 2^nd^ passage were selected for avoiding any chances of phenotype loss for all the experiments.

### *In vitro* and *in vivo* experimental design

To study the role of RT as chondroprotective *in vitro*, the chondrocytes were exposed to AGEs 50 μg/ml alone or was combined with RT at various concentrations (10, 20, 40 μM). The concentrations of RT were selected as per study earlier [26]. The control group of chondrocytes were not treated with RT, only the medium was changed. For studying the mechanism involved for role of RT mediated chondroprotective effect, the chondrocytes were exposed to AGEs (50 μg/ml) for 2 hours and the concentration of RT was 20 μM. In experiments involved with any functional changes in cells such as levels of ECM or inflammatory markers the time of exposure with AGEs was up to 24 hours.

For *in vivo* study in mice, the mice were submitted to surgical procedure as discussed in section 2.2. post-surgery, the DMM + RT treated mice were given RT solubilized in corn oil (40 mg/kg) [27] and was given via oral route one time in a day for eight weeks, the mice of control group were given equal volume of water. The mice of all the groups after eight week of treatment protocol were sacrificed and the tissue cartilages of knee joint were isolated for studying histology.

### The cell viability assay

For cell viability studies the chondrocytes (5 × 10^5^ in each well) were exposed to varied concentrations of AGEs (10, 20, 50, 100 and 200 μg/ml) or RT (5, 10, 20,40 μM) for 24 hours in serum free media followed by evaluation of viability with the help of cell counting kit-8 (CCK-8) (ThermoFisher USA) as per provided instructions.

### ELISA assay

The levels of NO in media were evaluated by Griess reagent method. After the isolated chondrocytes were treated with RT or AGEs, the culture media was collected and was analyzed for expression of MMP13, collagen II, PGE2, IL-6, TNF-α, ADAMTS-5 and MMP-13 using ELISA kit (ThermoFisher USA) following the provided instructions.

### Western blot analysis

After the chondrocytes were exposed to various concentrations of RT or AGEs the chondrocytes were rinsed with PBS and the cells were lysed using mixture of ice cold Radioimmunoprecipitation assay buffer (RIPA) buffer and phenyl-methanesulfonyl fluoride (1 mM), the cell lysate was obtained by centrifugation at 5000 rpm for 10 min at 4° C using a cooling centrifuge. The protein content was estimated using protein estimation kit (Sigma Aldrich USA). Protein (40 ng) was separated on sodium dodecylsulfate-polyacrylamide gel electrophoresis (SDS-PAGE) and was then transferred to PVDF membranes. The membranes were blocked using non-fat milk (5%) for 2 hours and were incubated with I^ry^ antibodies for iNOS (1:500), p65 (1:500), COX-2 (1:500), IκBα (1 : 500), p-JNK (1 : 500), JNK (1 : 500), p-p38 (1:500), p38 (1:500), Lamin B1 (1 : 500) and GAPDH (1:500), TBST (2%) was used as diluent, the blots were incubated at 4° C for 12 hours. The blots were again exposed to II^ry^ antibodies for 2 hours at 25° C, the membranes were the rinsed with TBST and viewed by electro-chemiluminescence, the intensity of the blots was measured using Image pro software.

### Immunofluorescence analysis

For analyzing the ADAMTS-5 and collagen II staining, the chondrocytes were transferred to glass petri-dish and plated with AGEs (50 μg/ml) or with AGEs+ RT (20 μM) for 24 hours. For evaluating the staining of p65 the time of exposure for AGEs was decreased to 120 minutes. Then the chondrocytes were treated with RT or AGEs and rinsed with phosphate buffered saline thrice and then treated with 400 μL paraformaldehyde (4%) for 10 minutes at 25° C the cells were then treated with Triton X 100 for enhancing the permeability. The chondrocytes were then added with bovine serum albumin (5%) at 25° C for 1 hour for blocking the cells. The chondrocytes after washing with PBS were treated with primary antibodies along with collagen II, ADAMTS5 and p65 (1:500) diluted in PBS for 12 hours at 4° C. After this the cells were then maintained with Alexa Fluor®488 labeled conjugated II^ry^ antibody for 60 minutes at 25° C followed by labeling with DAPI for 5 minutes. The pictures were recorded with the help of fluorescence microscope.

### Molecular docking studies

The 3D structure of ligand Rutin was built using ChemDraw (PerkinElmer, USA), the 3D crystal structure of p65, JNK and p38 were downloaded from protein data base. All the structures were minimized for energy with the help of PyMol (Version 1.7.6) (Schrodinger Inc., USA). The charges were added to the structures (Kolmann charges). The grid for each docking experiment between protein and ligand were built and docking was done with the help of Autodock MGL tools (The Scripps Research Institute, USA). The criteria for docking were based on interaction of proteins with ligand with minimal energy. The docking interactions and images were obtained and viewed using PyMol (Schrodinger Inc., USA).

### Histopathologic analysis

The histopathological analysis was done for the knee joints of OA mice treated with RT. The mice after receiving the treatments were anesthetized and sacrificed, knee joints were harvested and fixed using paraformaldehyde (4%). The knee joints were treated with formic acid (20%) for the process of decalcification and then embedded in paraffin. The tissue slides were submitted to staining with Safranin O-fast green. The slides were analyzed by experienced histopathologist of university, for evaluating the cellular and bone morphology, the observations were graded with Osteoarthritis Research Society International (OARSI) scoring system [28].

### Statistics

The results were calculated and were presented as mean ±SD. The statistics was performed using Graphpad prism software. The data were submitted to one-way analysis of variance (ANOVA) and then for comparison of groups (treated and control) by Tukey’s test. Kruskal–Wallis H test was used for calculating the Nonparametric data such as the OARSI scoring results. The value of P<0.05 were considered significant.

### Data availability

The data will be made available upon genuine request.

### Ethics declaration

The animal protocols were approved by the animal ethical review board of The Second Affiliated Hospital of Nanchang University, China. All the protocols of human experimental study were done in accordance guidelines from Declaration of Helsinki and were approved by human ethical review board of The Second Affiliated Hospital of Nanchang University, China. Informed consent was obtained from participating patients.

## RESULTS

### Rutin had no cytotoxic effect on human chondrocytes

**Figure 1:**
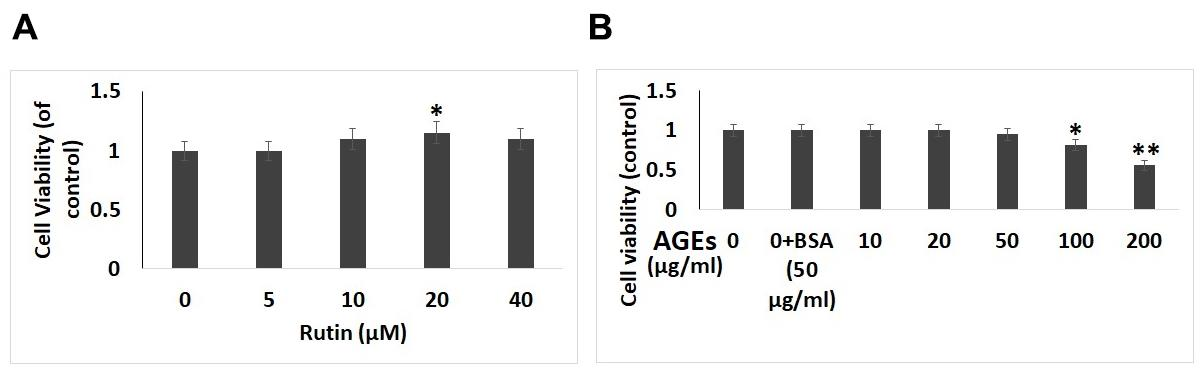
**Effect of AGEs and Rutin on viability of chondrocytes.** (**A**) Effect of various concentrations of Rutin on viability of chondrocytes. (**B**) Effect of exposure of AGEs on cell viability at various concentrations. The results are mean ± SD. *P<0.05, **P<0.01 compared to control.

The effect of RT as well as AGEs [29] on viability of isolated chondrocytes was evaluated by performing the CCK-8 assay. The results suggested that (Figure 1A), RT do not suppress the viability of chondrocytes post 24 hours of treatment at 5, 10, 20 and 40 μM suggesting that RT demonstrated no cytotoxic effect on chondrocytes at the selected doses and exposure time. We selected the 10, 20 and 40 μM concentrations of RT and duration of 24 hours for our experiments ahead. However, it was observed that AGEs at concentration of 100 and 200 μg/ml for 24 suppressed the viability of chondrocytes suggesting the cytotoxic effect of AGEs (Figure 1B).

### Rutin prevents AGE-induced inflammatory reactions in chondrocytes

**Figure 2:**
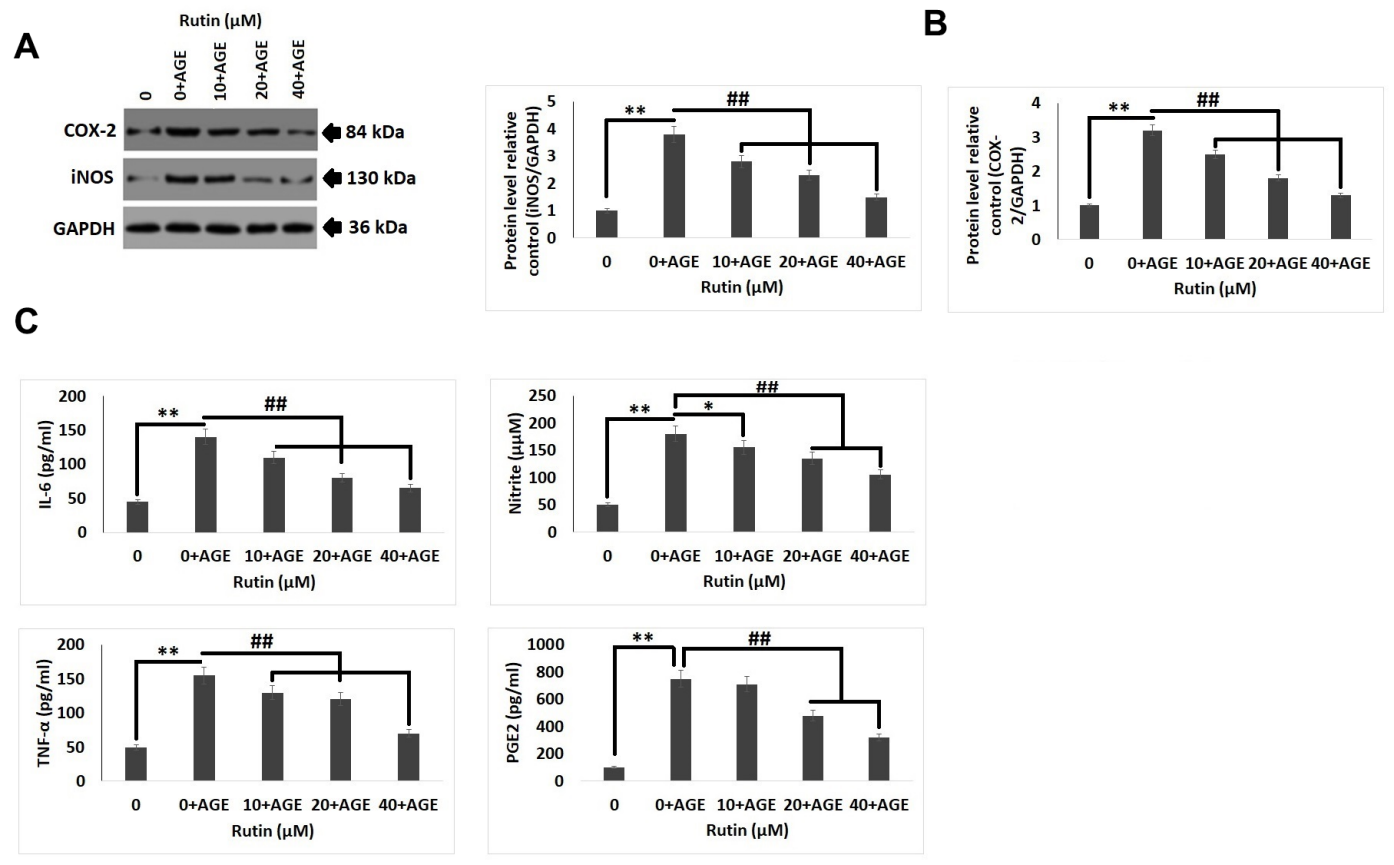
**Rutin ameliorates AGE-induced inflammatory reaction in chondrocytes.** (**A**, **B**) Western blot analysis for expression levels of COX-2 and iNOS in chondrocytes exposed AGE and various concentrations of Rutin. (**C**) Effect of treatment of Rutin on levels of IL-6, Nitrite, TNF-α and PGE2 at various concentrations. The results are mean ± SD. **P<0.01 compared to control, ##P<0.01 compared to AGE alone treated chondrocytes.

To study the anti-inflammatory activity of RT in human chondrocytes exposed to AGE, levels of inflammatory markers were evaluated by ELISA and immunoblotting analysis. The levels of COX-2 and iNOS (Figure 2A, 2B) were found to be increased after the chondrocytes were exposed to AGEs, it was also observed that exposure to RT reversed the levels of iNOS and COX-2 with increasing dose. The results of ELISA analysis suggested that, the treatment of RT decreased the AGE mediated up-regulation of IL-6, NO, TNF-α, and PGE-2 in a dose dependent manner (Figure 2C). The findings suggested that RT suppressed the mediators of inflammation and showed the anti-inflammatory response.

### Rutin alleviates ECM degradation in AGE exposed chondrocytes

**Figure 3:**
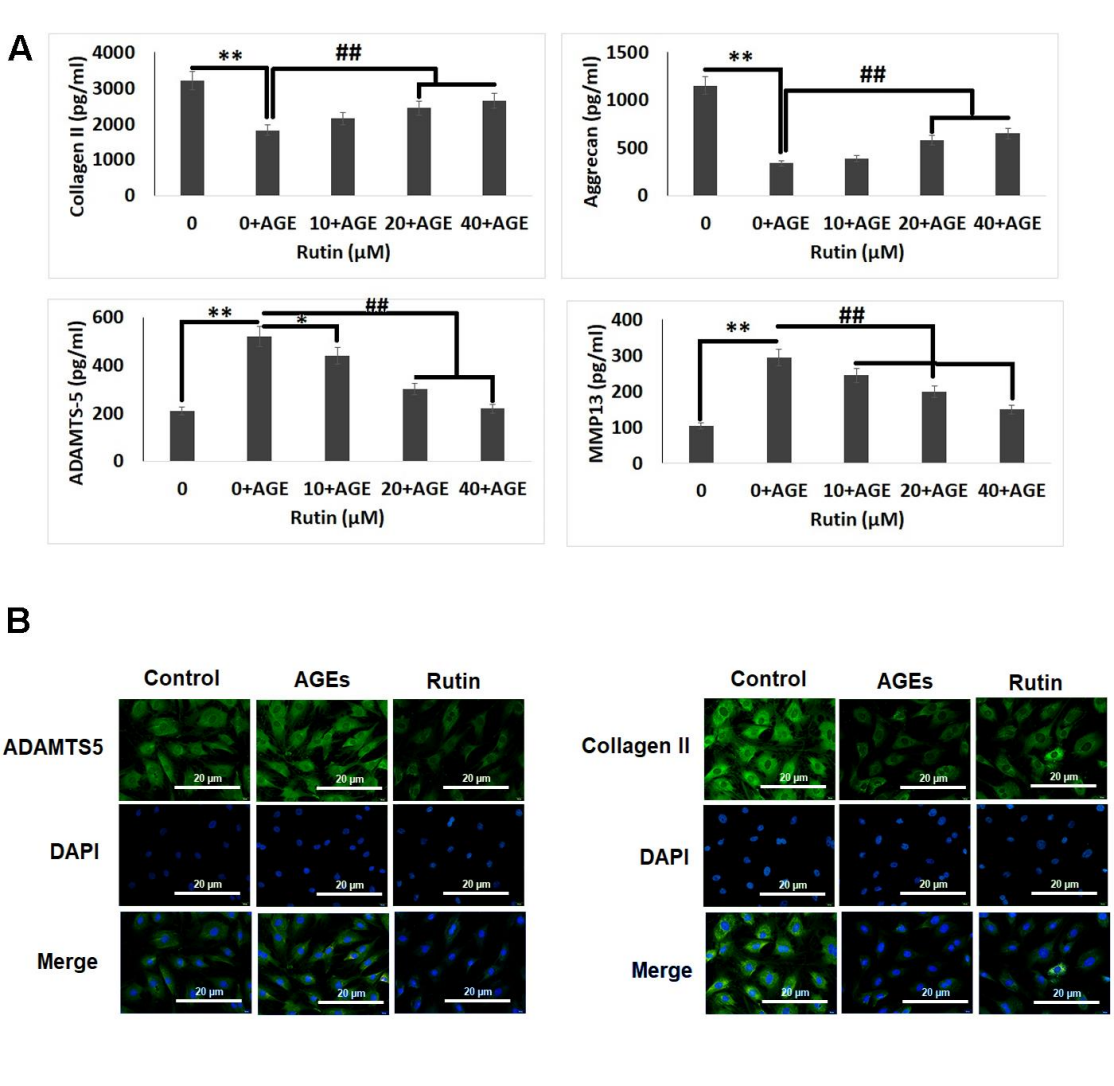
**Effect of Rutin on AGE-induced ECM degradation in chondrocytes.** (**A**) ELISA analysis for effect of Rutin treatment on levels of Collagen II, Aggrecan, ADAMTS-5 and MMP13. (**B**) Immunofluorescence along with DAPI staining analysis for detection of ADAMTS-5 and collagen II in chondrocytes. The results were mean ± SD. **P<0.01 compared to control, ##P<0.01 compared to AGEs alone treated chondrocytes.

Degradation and synthesis of extra cellular matrix (ECM) is regarded as an important product in chondrocytes which is used to evaluate the chondrocyte function [30]. The results of ELISA analysis in our study suggested that AGEs exposure resulted in decreased levels of aggrecan and collagen II but caused up-regulation of MMP-13 and ADAMTS-5. The exposure of RT showed a reversed trend in the levels of collagen II, aggrecan, ADAMTS-5 and MMP-13 in a dose dependent manner (Figure 3A). In addition to this, the results of immunofluorescence staining were parallel to the findings of ELISA assay (Figure 3B). The outcomes of the experiment suggested that RT could increase the protein levels of ECM degradation.

### Rutin suppresses AGE-induced trigger of the NF-κB pathway proteins

**Figure 4:**
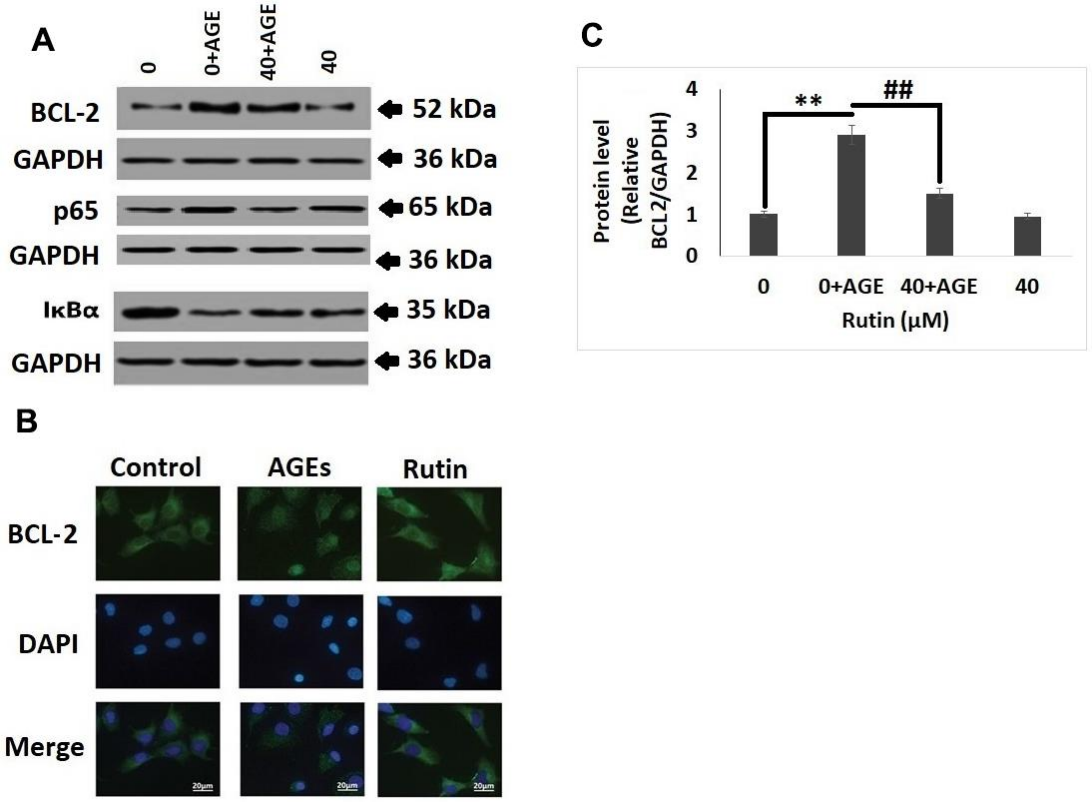
**Rutin suppresses AGE mediated induction on the NF-κB pathway proteins.** (**A**) Western blot analysis for expression of NF-κB pathway proteins BCL-2, p65 and IκBα in nuclei of AGEs chondrocytes treated with Rutin. (**B**) Immunofluorescence along with DAPI staining for studying the nuclei translocation of BCL-2. (**C**) Relative protein levels of BCL-2 in AGEs-induced chondrocytes treated with Rutin. The results were mean ± SD. **P<0.01 compared to control group, ##P<0.01 compared to AGEs alone treated group.

In elucidate the pathway involved behind the protective effect of RT in chondrocytes, western blot analysis was done for studying the expression of NF-κB associated genes (p65, IκBα and BCL-2). It was observed that AGEs induced the expression of BCL-2, however the treatment of RT inhibited this (Figure 4A, 4B). It was also observed that treatment of RT alone in chondrocytes devoid of AGEs did not showed any significant effects (Figure 4C). In addition to these results of immunofluorescent staining suggested that treatment of RT inhibited the RT induced nucleus BCL-2 fluorescence intensity which was in agreement to the findings of western blot analysis (Figure 4B).

### Rutin inhibits AGE-mediated MAPK cascade protein in chondrocytes

**Figure 5:**
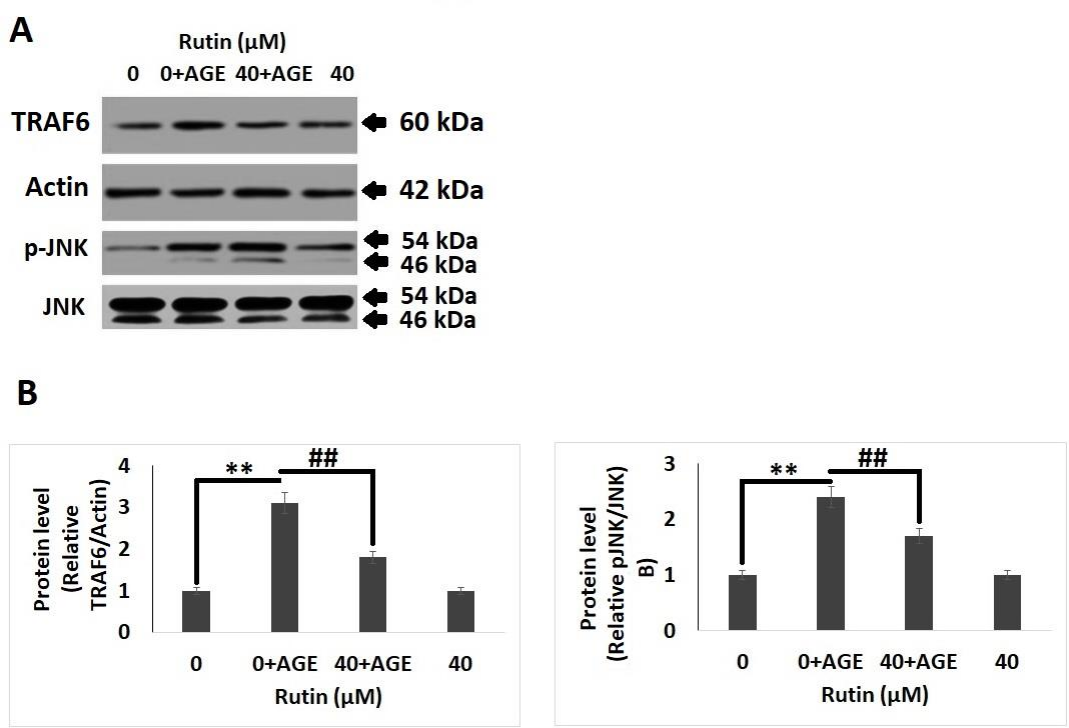
**Rutin inhibits AGE-induced MAPK pathway proteins in chondrocytes.** (**A**) Western blot analysis showing protein expression of TRAF6 and p-JNK. (**B**) Quantitative results showing relative expression of TRAF6 and JNK. The results were mean ± SD. **P<0.01 compared to control group, ##P<0.01 compared to AGEs alone treated group.

Mitogen-activated protein kinase (MAPK) cascade is associated with AGE-induced inflammation, oxidative stress and degeneration of ECM in chondrocytes. It was found that exposure to AGEs for 2 hours resulted in increased phosphorylation of JNK and levels of TRAF6, however treatment of RT decreased the phosphorylation of JNK and levels of TRAF6. It was also observed that treatments of RT alone do not showed a significant change in levels of p-JNK and TRAF6 compared to control group (Figure 5A, 5B).

### *In silico* molecular docking analysis

**Figure 6:**
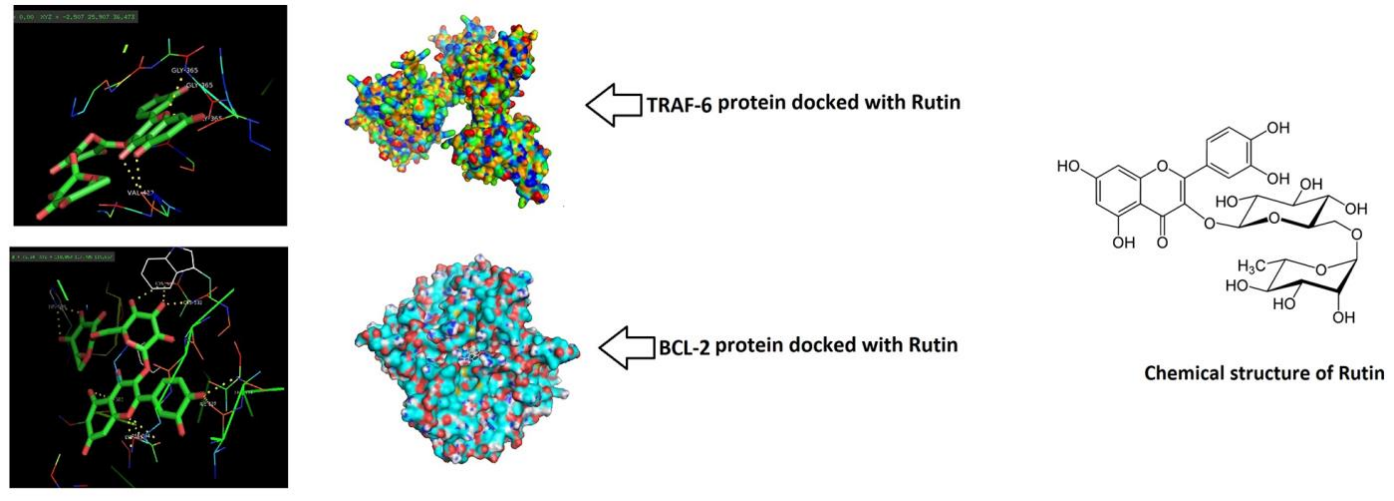
**Rutin showed potential binding with TRAF-6 and BCL-2 proteins.** Docking study was done and the surface solid pose showed potential binding of Rutin with TRAF-6 and BCL-2. The Ligand (Rutin) was majorly encapsulated in both the protein structures.

To provide concrete to the findings of study, molecular docking study was done to confirm the binding potential of RT with MAPK and the NF-κB cascade proteins. RT showed higher affinity for TRAF-6 and BCL-2 but poor binding with JNK the energies were -9.8 and -9.49, -2.5 kcal/mol respectively (Table 1). The surface model showed local interaction between TRAF-6, BCL-2 and JNK protein residues and the ligand RT. Due to poor interaction between JNK and RT the protein was not selected for detailed interaction studies. With TRAF-6 the ligand RT showed binding at the GLY-365 and VAL-412 fragments. With BCL-2 it was found that RT interacted with CYS-532, ILF-527, HIS-539 and LYS-483 fragments. Also, the solid structure of protein showed that the ligand RT was encapsulated majorly within the structure of proteins (Figure 6). The findings clearly suggested that MAPK and NF-κB pathways may be responsible involving the TRAF-6 and BCL-2 genes which might contribute for the ameliorative potential of RT in osteoarthritis induced chondrocytes.

**Table 1:** Results of docking parameters showing binding energies for affinity between the ligand (Rutin) and the protein TRAF-6 and BCL-2.

**TRAF-6**			
**Mode**	**Affinity (kcal/mol)**	**Distance from rmsd l.b.**	**Best mode rmsd u.b.**
1	-9.8	0.000	0.000
2	-9.8	13.188	13.162
3	-8.7	21.681	25.294
4	-7.8	11.494	12.801
5	-7.2	16.842	17.651
6	-6.9	14.713	13.197
7	-6.3	15.543	15.626
8	-4.2	24.641	23.557
9	-4.1	21.146	21.192

1	-9.49	0.000	0.0000
2	-8.1	24.221	23.461
3	-7.8	14.124	13.151
4	-6.9	16.391	15.745
5	-6.2	17.155	17.175
6	-5.5	19.415	18.188
7	-5.3	14.143	14.111
8	-4.4	17.122	16.944
9	-3.4	14.547	14.321

### RT protects from development of OA in experimental mice

**Figure 7:**
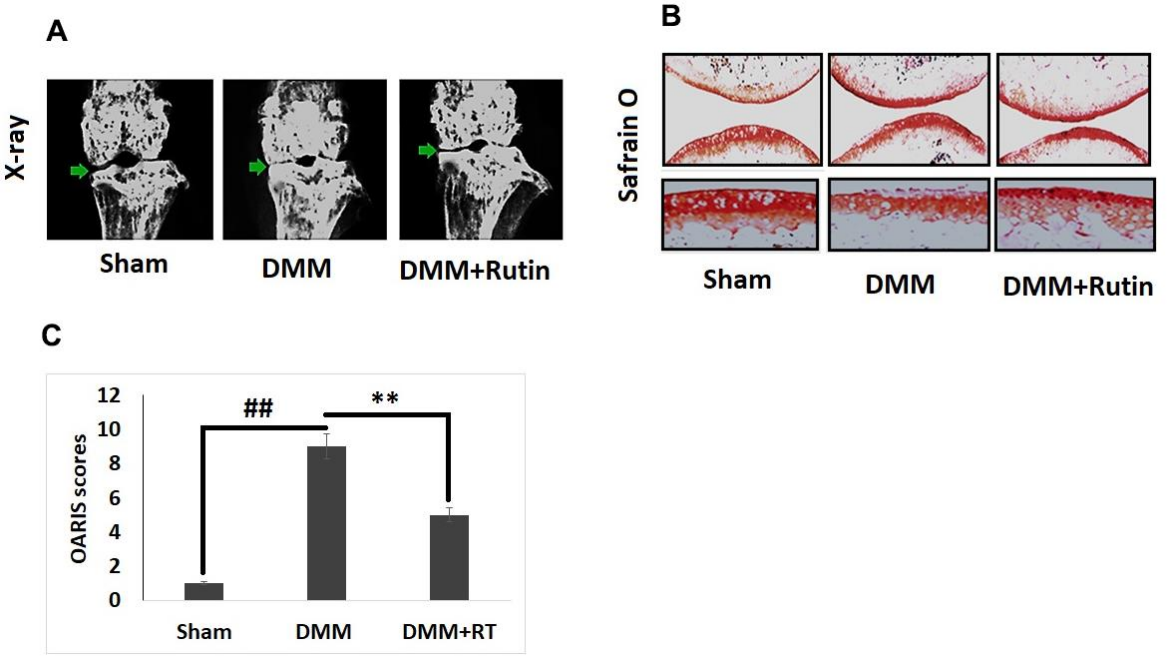
**Rutin ameliorates the development of OA in DMM mice.** (**A**) X-ray analysis showing knee of mice under various treatment groups. The OA mice showed narrowing of joint space and calcification of cartilage, however treatment of Rutin improved the joint space and also reduced the cartilage calcification. (**B**) S-O staining analysis of cartilage tissues in various treatment groups. (**C**) OARIS scoring of cartilage tissues of various treatment groups. The results are mean ± SD. ##P<0.01 compared to sham group, **P<0.01 compared to DMM induced mice.

For *in vivo* studies, we established the DMM mouse model of OA for studying the protective effect of RT against development of OA, followed by treatment of RT by oral gavage route once in a day for next eight weeks. The knee joints of the mice were evaluated by x-ray imaging (Figure 7A). The X-ray images demonstrated that the DMM mice had narrow joint space and also had calcification at the surface of cartilage compared to the sham group mice, treatment of RT improved the joint space and also reduced the calcification on the cartilage joints. The cartilages were submitted to safranin O staining for studying the histological changes (Figure 7B). The results of Safranin O staining suggested that the OA induced mice had rough cartilage surface with significant loss of proteoglycans, hypocellularity of chondrocytes with high OARSI score compared to the sham operate mice (Figure 7C). It was observed that the high OARSI score was decreased in mice treated with RT.

## DISCUSSION

Osteoarthritis (OA) is a chronic degenerative disorder of joints characterized by presence chronic pain with insufficient joint activity with limited options for preventive therapy [31]. The current treatment approaches involve use of non-steroidal anti-inflammatory drugs (NSAIDs) which are only helpful in countering the symptoms preventing the aggravation of OA but use of excessive NSAIDs have side effects such as liver and kidney damage also leads to severe cardiac issues [32]. Hence, investigation for agents with lesser side effects is always demanding in search for treating OA. RT is a flavonoid which is widely distributed in nature in fruits, vegetables and cereals. The flavonoid RT possesses range of biological activities such as anti-inflammatory, antioxidant and anti-infective [33]. However, role of RT, a potential flavonoid in joint degenerative disorder still remains unexplored, in this study we confirmed that RT suppressed the AGE-induced levels of COX-2-PGE-2 and iNOS-NO. The treatment also inhibited the levels of inflammatory factors such as MAPK and NF-κB pathways. Exposure of RT in AGEs-induced chondrocytes showed chondroprotective activity by inhibiting the degeneration of aggrecan and collagen II, alleviating levels of ADAMTS and MMP. In addition to this, results of *in vivo* analysis suggested that RT attenuated the development of OA.

In the development and progression of OA, development of inflammation and senescence are the prime factors, still the specific factor responsible remains a point of concern [18]. Studies have confirmed that in articular cartilages presence of AGE is directly correlated to age [34, 35]. It was reported that the levels of AGEs in subjects aging less than 20 years were significantly lower compared to those aging more than 60 years [34]. In addition to this, levels of AGEs are on higher side in diabetic subjects with OA [36]. Hence number of studies have emerged which have used AGEs as agent for mimicking OA *in vitro* [12, 37–39]. In the present work, we evidenced that AGEs at dose of 50 μg/ml do not affected the viability of chondrocytes as observed by CCK-8 assay. In a study earlier, AGEs at high concentration in chondrocytes leads to apoptosis whereas at lower concentrations i.e of 50 μg/ml AGEs causes inflammation in the cartilage tissues. Accumulation of AGE results in an inflammatory reaction leading to degeneration of ECM which is characteristic feature of chondrocytes. Number of studies have shown that exposure of chondrocytes to AGE resulted in activation of MAPK and NF-κB pathway and also lead to overexpression of COX-2, MMPs, IL-8, IL-6, ADAMTS and TNF-α leading to degeneration of ECM [14, 15]. NF-κB pathway is one of the important pathways associated with OA. IκBα, p65 and BCL-2 are proteins of NF-κB pathway [40]. Studies earlier have suggested that BCL-2 leads to over expression of inflammatory mediators such as IL-6, PGE-2, TNF-α, iNOS and COX-2 [41]. PGE-2 is produced by ADAMTS and MMP induced cartilage degeneration and COX-2 [42]. TRAF-6 is one the direct activator of MAPK pathway [29, 43]. The findings of present study suggested that RT significantly suppressed the overexpression of COX-2, NO, iNOS and PGE2 at both protein and mRNA levels in human chondrocytes exposed to AGEs. The treatment of RT also inhibited the levels of IL-6 and TNF-α. Further, exposure to RT ameliorated AGEs overexpression of TRAF-6 and BCL-2 the findings were in agreement to earlier report in which RT suppressed the expression of TRAF-6 and BCL-2 in inflammatory conditions [26, 27]. The outcomes of molecular docking analysis suggested that RT had potential binding with TRAF-6 and BCL-2.

We in our study also developed and established the mouse model of DMM for screening whether RT could show protective effect in OA *in vivo*. The mouse DMM model presented cartilages with rough surface, chondrocytes with hypo-cellularity with high OARSI scores. However, it was observed that the treatment with RT corrected these features in DMM mice with significant decrease in OARSI scores.

In conclusion, the present study suggested that RT suppressed the AGE-induced inflammatory reaction and also ECM degradation by suppressing the MAPK and NF-κB cascade in human chondrocytes. It was also evidenced that upon oral treatment of RT in DMM mice inhibited the development of OA. The findings demonstrated that RT could be a potential molecule for treating OA. However clinical studies are required for establishing efficacy of RT in humans.
